# Clinical Effectiveness and Cost Implications of a Community Psychosocial Rehabilitation Service for Severe and Persistent Mental Illness in Nova Scotia, Canada

**DOI:** 10.1192/bjo.2024.460

**Published:** 2024-08-01

**Authors:** Mahmoud Awara

**Affiliations:** Dalhousie Medical School, Halifax, Canada

## Abstract

**Aims:**

People with severe and persistent mental illness (SPMI) present unique challenges in mental healthcare due to the enduring nature and complexity of their conditions. The study focuses on evaluating the clinical effectiveness and cost implications of a multidisciplinary community psychosocial rehabilitation team catering to individuals with SPMI in Nova Scotia, Canada. The investigation seeks to contribute valuable evidence to the limited literature on community psychosocial rehabilitation in the Canadian context.

**Methods:**

The study adopts a retrospective approach, analyzing data from patients referred to community rehabilitation between 2016 and 2017. The assessment centers on the year before and after patient engagement with the community rehabilitation team. Clinical effectiveness is evaluated through measures of inpatient service use (admissions, length of stay) and emergency department (ED) visits. The Canadian billing system of Medical Service Insurance (MSI) is employed to examine the cost of acute service utilization.

**Results:**

Results demonstrate a statistically significant reduction in mean admission rates and length of stay in the post-rehabilitation year compared with the pre-rehabilitation period. A substantial percentage of patients experienced no inpatient admissions or ED visits in the post-rehabilitation year. The analysis reveals a significant net reduction in hospital days, translating into substantial cost savings. The findings highlight the potential economic benefits of community rehabilitation in the context of SPMI.

**Conclusion:**

The study suggests that community rehabilitation contributes positively to the clinical outcomes of individuals with SPMI, showcasing reduced inpatient service use and associated costs. The findings underscore the importance of further research into community psychosocial rehabilitation in the Canadian setting and emphasize the economic implications essential for demonstrating the efficiency of mental healthcare services.

